# Evaluation of Lipid Profile Modulation by Berberis asiatica, Withania somnifera, and Their Synergy in Type 2 Diabetic Wistar Rats

**DOI:** 10.7759/cureus.67974

**Published:** 2024-08-27

**Authors:** Devkumar D Tiwari, Vandana M Thorat, Prathamesh V Pakale, Sarika Patil, Dhanashri Chavan

**Affiliations:** 1 Department of Pharmacology, Krishna Vishwa Vidyapeeth (Deemed to be University), Karad, IND

**Keywords:** dyslipidemia, ashwagandha (withania somnifera), synergistic effects, herbal medicine, cardiovascular risk, lipid profile, berberis asiatica, diabetes mellitus type 2

## Abstract

Introduction

Type 2 diabetes mellitus (T2DM) is a chronic metabolic disorder characterized by insulin resistance and hyperglycemia, leading to complications such as dyslipidemia, which increases cardiovascular risks. Current treatments for dyslipidemia often have undesirable side effects. This study aims to evaluate the effects of *Berberis asiatica* (*BA*), *Withania somnifera* (*WS*), and their combination in the ratio of 1:1 on the lipid profile in T2DM-induced Wistar rats. Additionally, the study investigates the potential synergistic effects of these two herbs.

Materials and methods

Mature albino Wistar rats of both sexes were employed, weighing 150-250 g. Rats were obtained from the Central Animal House of Krishna Institute of Medical Sciences and kept under standard laboratory conditions. The study was conducted per the guidelines set by the Committee for Control and Supervision of Experiments on Animals (CCSEA). T2DM was induced using streptozotocin (STZ) and nicotinamide (NIC). Thirteen groups of rats were formed, including normal control (NC), diabetic control (DC), and various treatment groups received varying dosages of *BA*, *WS*, their polyherbal combination (PHC), and the conventional medications metformin (MET) and glimepiride (GLI). Lipid profiles were measured, and the data were analyzed using one-way ANOVA, followed by the Tukey-Kramer post-hoc test.

Results

The study revealed that both *BA* and *WS* showed statistically significant lipid-lowering effects in diabetic rats. The *BA*-treated groups displayed a statistically significant and considerable decrease in total cholesterol (TC) and low-density lipoprotein (LDL) levels compared to the DC group. Similarly, *WS*-treated groups also showed statistically significant reduced levels of TC and LDL, along with an increase in high-density lipoprotein (HDL). The PHC of *BA* and *WS* exhibited enhanced lipid-lowering effects compared to individual treatments. No significant differences in triglyceride (TG) levels were observed among the treatment groups.

Conclusion

*BA* and *WS*, individually and in combination, effectively modulate lipid profiles in T2DM rats. Their synergistic effects provide a promising alternative for managing dyslipidemia in diabetic patients. Further research is needed to determine the clinical consequences of these findings.

## Introduction

Type 2 diabetes mellitus (T2DM) is also known as non-insulin-dependent diabetes mellitus (NIDDM) or maturity-onset diabetes mellitus (DM). It is a chronic metabolic disorder characterized by insulin resistance and hyperglycemia [1,2] beyond glycemic control, leading to various complications, including dyslipidemia, which significantly contributes to an elevated risk of cardiovascular diseases [3]. Dyslipidemia, a common condition in diabetic patients, involves abnormal levels of lipids in the blood and is a significant risk factor for cardiovascular diseases [4]. Abnormalities in lipid profiles, including elevated triglycerides, reduced high-density lipoprotein (HDL), and altered low-density lipoprotein (LDL), play a crucial role in the etiology of diabetic cardiovascular complications (CVCs) [5]. Managing lipid profiles in diabetic patients is critical for preventing and avoiding CVCs and improving overall health outcomes [6]. Despite the various drugs available for the treatment of dyslipidemia on the market, they fail to provide an ideal solution and come with undesirable side effects such as myalgias (muscular aches), rhabdomyolysis (muscle breakdown), transaminitis (liver inflammation), liver failure, and an increased risk of DM [7].

The relationship between diabetes, lipid metabolism, and cardiovascular health warrants a growing interest in discovering natural organic substances that can modulate lipid profiles. Two such intriguing and promising alternative candidates are *Berberis asiatica* (*BA*) [8] and *Withania somnifera* (WS). Both plants have been in long-established conventional medicine systems, such as Ayurveda, the Indian conventional and traditional medical system like "Siddha" [9], and the Chinese traditional medicine system, for their potential antidiabetic and other medicinal properties [10].

Approximately 60% of the global population uses traditional medicines originating naturally from medicinal plants [11]. Natural products, along with traditional medicine, have attracted a lot of attention for their potential in managing diabetes and its associated complications due to their phytochemicals, showing promise for prevention and/or control for better management [12]. In our study, *BA* and *WS* were selected as medicinal herbs with promising antidiabetic and lipid-lowering properties [13].

*BA*, commonly known as Indian barberry, has been traditionally utilized in the science of Ayurveda for its various pharmacological effects, including antidiabetic, anti-inflammatory, and antioxidant properties. Studies have shown that berberine, an active component of *BA*, can improve lipid metabolism and reduce blood glucose levels [14]. *WS*, also known as Ashwagandha or "Indian ginseng," is another widely used herb in traditional medicine. It is renowned for its adaptogenic, anti-inflammatory, and hypoglycemic effects. Research suggests that *WS* can modulate lipid profiles and enhance insulin sensitivity, making it beneficial in managing T2DM [15].

Various studies have explored the synergistic effects of combining multiple herbs, revealing enhanced therapeutic benefits compared to single-herb treatments [16]. This study aims to evaluate the effects of *BA, WS*, and their combination on the lipid profile in Wistar rats induced with T2DM. Understanding the potential synergy between these two herbs allows us to identify and develop more effective techniques and strategies for managing dyslipidemia in diabetic patients.

## Materials and methods

The dried ethanolic root extract of *BA* and *WS* was procured from Natucare India Pvt. Ltd., Mumbai, India, and Bhagwati Herbal and Healthcare Pvt. Ltd., Vapi, India, in pure powder form. Streptozotocin (STZ) and nicotinamide (NIC) were sourced from Sisco Research Laboratories Pvt. Ltd., Mumbai, India. Metformin (MET) and glimepiride (GLI) were sourced from Smruthi Organic Limited, Solapur, India, in pure powder form [17].

Experimental animal

Animal Selection and Source

This study involves mature (six to eight weeks old) albino Wistar rats of both sexes, each weighing 150-250 g. These animals were obtained from the central animal house of Krishna Institute of Medical Sciences (KIMS), Krishna Vishwa Vidyapeeth (KVV), Satara, India.

Housing and Care Conditions

The rats were housed in a controlled environment under standard laboratory conditions, including 12 hours of a light/dark cycle with a temperature maintained between 27 °C and 37 °C. Throughout the study, the animals had unrestricted access to food and water.

Ethical Approval and Compliance

The study was conducted following ethical standards, with necessary approvals obtained from the Institutional Ethics Committee (IEC) of KVV (IEC approval no. 385/2020-2021) on August 25, 2021, and the Institutional Animal Ethics Committee of KVV (Deemed to be University), Reg. No. 255/PO/REBi/S/2000/CPCSEA (IAEC approval no. IAEC/KIMS/2021/16) on November 20, 2021. All the experimental procedures were carried out according to guidelines set forth by the Committee for Control and Supervision of Experiments on Animals (CCSEA) in the Central Animal House, KVV [17-19].

Acute oral toxicity assessment

The acute toxicity evaluation was conducted in adult Wistar rats following the "limit dose" method as outlined in the Organization for Economic Co-operation and Development (OECD) Guideline No. 240 [20]. A test procedure began administering an initial starting dose of 2000 mg/kg BW. Before the administration, the rats were fasted overnight. The following day, dried ethanolic root extracts of the plants BA and WS were given orally at a 2000 mg/kg BW dosage. Post-administration, the animals were closely monitored for a continuous period of three hours, focusing on general psychological behavioral and neural and autonomic characteristics. The monitoring continued at 30-minute intervals for an additional three hours, and the animals were finally observed for any signs of mortality over a period extending from 24 hours to 14 days [20].

Limit test execution

In alignment with OECD guidelines, it was ensured that the cumulative dose of the PHC would not exceed 2000 mg/kg BW, which is considered the upper limit dose to assess acute toxicity. The second animal received the identical dose as the initial one, and both survived after being given the upper limit dose. A total of three animals were administered the limiting dose, and as no fatalities occurred, the three additional animals of the opposite sex were tested at the limited dose level. The absence of lethality in all tested subjects led to the conclusion of the test.

Experimental design

**Figure 1:**
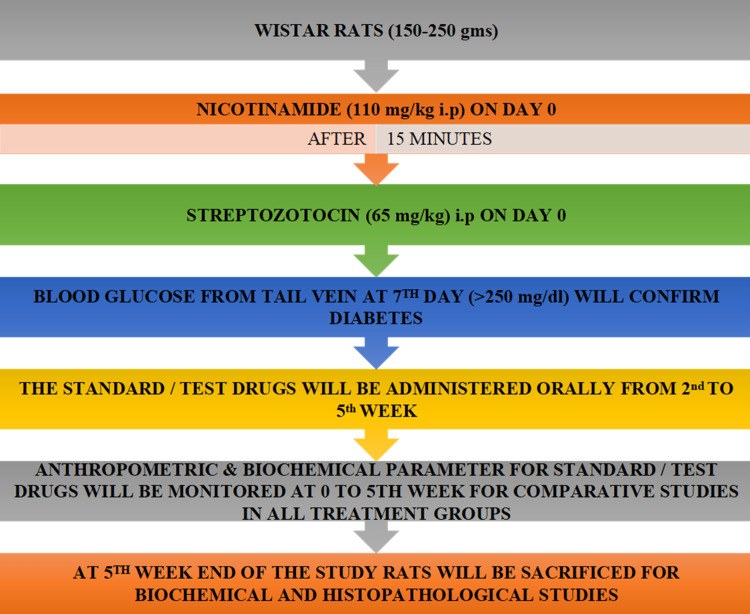
Detailed methodology Gms: grams; ml: milliliter; mg: milligram; kg: kilogram; dl: deciliter; i.p: intraperitoneal

A total of 78 rats were used in 13 different groups, with six rats in every group for the experiments. Table 1 presents all 13 groups of distribution of rats with their specific drugs, dosage forms, and route of administration. Each group consists of six animals. A detailed methodology of the study is also presented in Figure 1 [17].

**Table 1:** Experimental groups and treatment protocol NC: normal control; DC: diabetic control; BA: Berberis asiatica; WS: Withania somnifera; PHC: polyherbal combination; MET: metformin; GLI: glimepiride

Group No.	Group Name	Extract/Drugs	Dose and Route (Orally) mg/kg
1	NC	Distilled water	10 mL/kg
2	DC	Distilled water	10 mL/kg
3	BA 250	Dried ethanolic root extract of BA	250
4	BA 500	Dried ethanolic root extract of BA	500
5	BA 1000	Dried ethanolic root extract of BA	1000
6	WS 250	Dried ethanolic root extract of WS	250
7	WS 500	Dried ethanolic root extract of WS	500
8	WS 1000	Dried ethanolic root extract of WS	1000
9	PHC 250	Dried ethanolic root extract of BA + WS	125 + 125
10	PHC 500	Dried ethanolic root extract of BA + WS	250 + 250
11	PHC 1000	Dried ethanolic root extract of BA + WS	500 + 500
12	MET	Metformin (standard)	250
13	GLI	Glimepiride (standard)	10

Drug administration and sources

Each test drug used in this experiment was dissolved in distilled water to ensure proper administration. The solutions were freshly prepared each day to maintain potency and were administered orally to the rats starting from the seventh day and continuing daily until the conclusion of the experiment on the 35th day. The dosages for both the pharmaceutical drugs and the herbal extracts were carefully determined based on prior research conducted within our laboratory as well as from established literature sources [17].

Development of T2DM and sample collection

T2DM was experimentally induced in the rats through intraperitoneal (i.p.) administration of STZ at a dose of 65 mg/kg BW dissolved in physiological saline. This was carried out 15 minutes after an i.p. injection of NIC at a dose of 110 mg/kg BW [19]. The rats were observed and monitored closely over the course of seven days after the STZ-NIC injection procedure. Random blood glucose (RBG) levels were measured using the tail vein prick method with the help of BeatO CURV (standard portable digital android glucometer, Kaynes Technology, New Delhi, India). The rats that exhibited RBG levels exceeding 250 mg/dL on the seventh-day post-injection were classified as diabetic and subsequently included in this study [10]. At the conclusion of the experimental period, the rats were humanely euthanized using an overdose of ketamine. Blood samples were collected directly from the heart via the heart puncture technique after sacrificing the rat. The collected blood samples were then transferred into plain tubes for subsequent biochemical analyses, such as the estimation of lipid profiles(HDL, LDL, TG, and TC) [17]. For the calculation of LDL, the Friedewald equation was used, i.e., LDL = TC − HDL − TGs/5 [21].

Statistical analysis

The data were evaluated using a one-way analysis of variance (ANOVA) to determine the differences among the experimental groups. Following ANOVA, a post-hoc Tukey-Kramer test was conducted to pinpoint specific group comparisons that exhibited statistically significant differences. The results for blood glucose levels across all groups are expressed as mean ± standard deviation (SD). A significance threshold was set at P ≤ 0.05 for all statistical tests. The analyses were conducted using IBM SPSS Statistics for Windows, Version 18 (Released 2009; IBM Corp., Armonk, New York).

## Results

**Figure 2:**
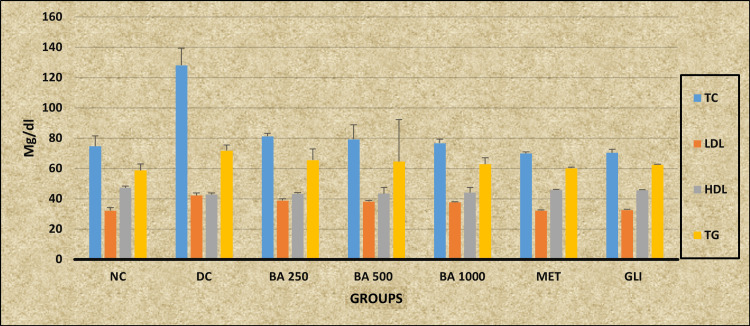
Graphical representation of the effect of Berberis asiatica on the lipid profile of rats NC: normal control; DC: diabetic control; BA: Berberis asiatica; MET: metformin; GLI: glimepiride; TC: total cholesterol; LDL: low-density lipoprotein; HDL: high-density lipoprotein; TG: triglycerides

Ordinary ANOVA followed by the Tukey-Kramer multiple comparison test (post hoc) revealed statistically significant differences in TC, LDL levels (P < 0.0001), and HDL levels (P = 0.0050), whereas no statistically significant differences were observed in TG levels (P = 0.5381) in all the study groups (NC, DC, *BA* 250, *BA* 500, *BA* 1000, MET, and GLI). A post-hoc test revealed a statistically significant decrease in TC and LDL levels in all the study groups (NC, *BA* 250, *BA* 500, *BA* 1000, MET, and GLI) when compared with the DC group (P < 0.001). No statistically significant difference is shown by all the study groups (*BA* 250, *BA* 500, *BA* 1000, MET, and GLI) when compared to the NC group (P > 0.05) except the DC group, which shows statistically significant differences (P < 0.001). It also revealed a statistically significant increased level of LDL in DC, *BA* 250, *BA* 500, and *BA* 1000 when compared with the NC group and the standard control (MET and GLI) group (P < 0.001). There were no statistically significant differences observed in the LDL levels of the NC, MET, and GLI groups when compared with each other (P > 0.05). A post-hoc test shows no statistically significant difference in HDL and TG levels in all study groups (DC, *BA* 250, *BA* 500, *BA* 1000, MET, and GLI) (P > 0.05), except the NC group, which shows a statistically significant higher level of HDL when compared with the DC group (P < 0.05). Whereas no statistically significant differences were observed in the TG level in all the study groups (NC, DC, *BA* 250, *BA* 500, *BA* 1000, MET, and GLI) (P > 0.05) (Table 2) (Figure 2).

**Table 2:** Effect of Berberis asiatica on the lipid profile of rats Data are expressed as mean ± SD, n = 6 in each group a: DC differs significantly from BA 250, BA 500, BA 1000, MET, and GLI groups; 1: P < 0.05; b: MET differs significantly from DC, BA 250, BA 500, BA 1000, and GLI groups; 2: P < 0.01; c: GLI differs significantly from DC, BA 250, BA 500, BA 1000, and MET groups; 3: P < 0.001 TC: total cholesterol; LDL: low-density lipoprotein; HDL: high-density lipoprotein; TG: triglycerides; NC: normal control; DC: diabetic control; BA: Berberis asiatica; MET: metformin; GLI: glimepiride; F: F-statistics; P: probability

Groups	NC (mg/dL)	DC (mg/dL)	BA 250 (mg/dL)	BA 500 (mg/dL)	BA 1000 (mg/dL)	MET (mg/dL)	GLI (mg/dL)	F	P
Test									
TC	74.67 ± 6.68^a3^	128.07 ± 11.26^b3c3^	81.17 ± 1.94^a3^	79.27 ± 9.4^a3^	76.67 ± 2.58^a3^	69.83 ± 1.17^a3^	70.33 ± 2.25^a3^	63.06	P < 0.0001
LDL	32.13 ± 1.9^a3^	42.15 ± 1.63^b3c3^	38.83 ± 1.17^a3b3c3^	38.16 ± 0.76^a3b3c3^	37.58 ± 0.44^a3b3c3^	32.17 ± 0.34^a3^	32.52 ± 0.40^a3^	75.805	P < 0.0001
HDL	47.33 ± 1^a1^	42.77 ± 0.92	43.3 ± 0.67	43.4 ± 4.1	44.01 ± 3.5	45.83 ± 0.3	45.67 ± 0.28	3.817	0.0050
TG	58.67 ± 4.3	71.67 ± 3.72	65.51 ± 7.4	64.51 ± 27.8	62.81 ± 4.14	60.1 ± 0.61	62.35 ± 0.36	0.8535	0.5381

**Figure 3:**
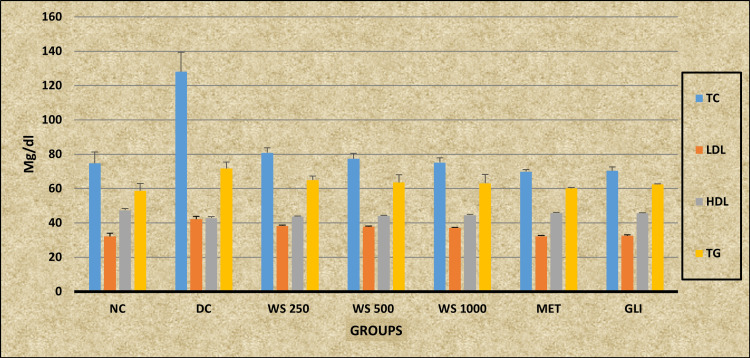
Graphical representation of the effect of Withania somnifera on the lipid profile of rats NC: normal control; DC: diabetic control; WS: Withania somnifera; MET: metformin; GLI: glimepiride; TC: total cholesterol; LDL: low-density lipoprotein; HDL: high-density lipoprotein; TG: triglycerides

Ordinary ANOVA followed by the Tukey-Kramer multiple comparison test (post hoc) revealed statistically significant differences in TC, LDL, HDL, and TG levels (P < 0.0001) in all the study groups (NC, DC, *WS* 250, *WS* 500, *WS* 1000, MET, and GLI). A post-hoc analysis revealed a statistically significant decrease in the TC and LDL levels in all the study groups (NC, *WS* 250, *WS* 500, *WS* 1000, MET, and GLI) when compared with the DC group (P < 0.001). *WS* 250 shows a statistically significant increased level of TC when compared with the MET and GLI groups (P < 0.05). Additionally, a statistically significant increased level of LDL in the study groups *WS* 250, *WS* 500, and *WS* 1000 is observed when compared with the MET, GLI, and NC groups (P < 0.001). A statistically significant increased level of HDL is observed by the study groups *WS* 500 (P < 0.01), WS1000, MET, GLI, and NC (P < 0.001) when compared to the DC group, whereas the *WS* 250 group shows no statistically significant differences in the HLD level in comparison with the DC group (P > 0.05). However, compared with the MET and GLI study groups, a statistically significant decreased HDL (P < 0.001) is observed. A post-hoc analysis also revealed a statistically significant decrease in the TG level in the study groups *WS* 250 (P < 0.05), *WS* 500, *WS* 1000 (P < 0.01), MET, GLI, and NC (P < 0.001) when compared with the DC group, whereas the DC group showed a statistically significant elevated TG level when compared with the NC, MET, and GLI groups (P < 0.001), the *WS* 500 and *WS* 100 (P < 0.01), and the *WS* 250 (P < 0.05) (Table 3) (Figure 3).

**Table 3:** Effect of Withania somnifera on the lipid profile of rats Data are expressed as mean ± SD, n = 6 in each group a: DC differs significantly from WS 250, WS 500, WS 1000, MET, and GLI groups; 1: P < 0.05; b: MET differs significantly from DC, WS 250, WS 500, WS 1000, and GLI groups; 2: P < 0.01; c: GLI differs significantly from DC, WS 250, WS 500, WS 1000, and MET groups; 3: P < 0.001 TC: total cholesterol; LDL: low-density lipoprotein; HDL: high-density lipoprotein; TG: triglycerides; NC: normal control; DC: diabetic control; WS: Withania somnifera; MET: metformin; GLI: glimepiride; F: F-statistics; P: probability

Groups	NC (mg/dL)	DC (mg/dL)	WS 250 (mg/dL)	WS 500 (mg/dL)	WS 1000 (mg/dL)	MET (mg/dL)	GLI (mg/dL)	F	P
Test									
TC	74.67 ± 6.68^a3^	128.07 ± 11.26	80.83 ± 2.86^a3b^^1^^c^^1^	77.33 ± 3.14^a3^	75.17 ± 2.64^a3^	69.83 ± 1.17^a3^	70.33 ± 2.25^a3^	87.268	P < 0.0001
LDL	32.13 ± 1.9^a3^	42.15 ± 1.63^b3c3^	38.3 ± 0.4^a3b3c3^	37.75 ± 0.37^a3b3c3^	37.17 ± 0.24^a3b3c3^	32.22 ± 0.34^a3^	32.52 ± 0.40^a3^	89.92	P < 0.0001
HDL	47.33 ± 1^a3^	42.77 ± 0.92	43.63 ± 0.33^b3c3^	44.14 ± 0.25^a2b3c2^	44.53 ± 0.44^a3b2c1^	45.83 ± 0.3^a3^	45.67 ± 0.28^a3^	40.555	P < 0.0001
TG	58.67 ± 4.3^a3^	71. 67 ± 3.72^b3c3^	64.96 ± 2.4^a1^	63.51 ± 4.5^a2^	63.05 ± 5.1^a2^	60.1 ± 0.61^a3^	62.35 ± 0.36^a3^	8.732	P < 0.0001

**Figure 4:**
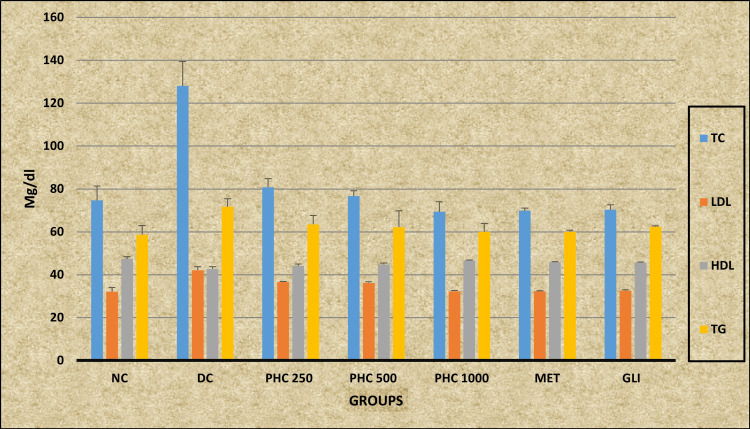
Graphical representation of the effect of the polyherbal combination on the lipid profile of rats NC: normal control; DC: diabetic control; PHC: polyherbal combination; MET: metformin; GLI: glimepiride; TC: total cholesterol; LDL: low-density lipoprotein; HDL: high-density lipoprotein; TG: triglycerides

Ordinary ANOVA followed by the Tukey-Kramer multiple comparison test (post hoc) revealed statistically significant differences in all the lipid profile parameters TC, LDL, HDL, and TG levels (P ≤ 0.0001) in all the study groups (NC, DC, PHC 250, PHC 500, PHC 1000, MET, and GLI). A post-hoc analysis revealed a statistically significant decrease in the TC and LDL levels in all the study groups (NC, PHC 250, PHC 500, PHC 1000, MET, and GLI) when compared with the DC group (P < 0.001). PHC 250 shows a statistically significant increased level of TC compared with the MET and GLI groups (P < 0.05). Additionally, a statistically significant high level of LDL is observed in the study groups PHC 250 and PHC 500 compared to the MET, GLI, and NC groups (P < 0.001). A statistically significant increased level of HDL is observed by all study groups (PHC 500, PHC 1000, MET, GLI, and NC) when compared to the DC group (P < 0.001), except PHC 250, whereas the PHC 250 group shows no statistically significant differences in the HLD level in comparison with the DC group (P > 0.05). However, compared with the MET and GLI study groups, a statistically significant low level of HDL (P < 0.01) is revealed. Post-hoc analysis also showed a statistically significant decreased level of TG in the study groups PHC 250 (P < 0.05), PHC 500 (P < 0.01), PHC 1000, MET, GLI, and NC (P < 0.001) when compared with the DC group (Table 4) (Figure 4).

**Table 4:** Effect of the polyherbal combination on the lipid profile of rats Data are expressed as mean ± SD, n = 6 in each group a: DC differs significantly from PHC 250, PHC 500, PHC 1000 MET, and GLI groups; 1: P < 0.05; b: MET differs significantly from DC, PHC 250, PHC 500, PHC 1000, and GLI groups; 2: P < 0.01; c: GLI differs significantly from DC, PHC 250, PHC 500, PHC 1000, and MET groups; 3: P < 0.001 TC: total cholesterol; LDL: low-density lipoprotein; HDL: high-density lipoprotein; TG: triglycerides; NC: normal control; DC: diabetic control; PHC: polyherbal combination; MET: metformin; GLI: glimepiride; F: F-statistics; P: probability

Groups	NC (mg/dL)	DC (mg/dL)	PHC 250 (mg/dL)	PHC 500 (mg/dL)	PHC 1000 (mg/dL)	MET (mg/dL)	GLI (mg/dL)	F	P
Test									
TC	74.67 ± 6.68^a3^	128.07 ± 11.26^b3c3^	80.83 ± 3.98^a3b^^1^^c^^1^	76.67 ± 2.5^a3^	69.33 ± 4.68^a3^	69.83 ± 1.17^a^^3^	70.33 ± 2.25^a3^	694.6	P < 0.0001
LDL	32.13 ± 1.9^a3^	42.15 ± 1.6^b3c3^	36.58 ± 0.35^a3b3c3^	36.23 ± 0.5^a3b3c3^	32.27 ± 0.41^a3^	32.22 ± 0.34^a3^	32.52 ± 0.41^a3^	83.54	P < 0.0001
HDL	47.33 ± 1.03^a3b1c2^	42.77 ± 0.92^b3c3^	44.04 ± 0.95^b2c2^	44.76 ± 0.72^a3^	46.58 ± 0.29^a3^	45.83 ± 0.3^a3^	45.67 ± 0.28^a3^	28.227	P < 0.0001
TG	58.67 ± 4.32^a3^	71.67 ± 3.72^b3c2^	63.48 ± 4.12^a1^	62.25 ± 7.46^a2^	60.09 ± 3.78^a3^	60.09 ± 0.61^a3^	62.35 ± 0.36^a2^	6.493	0.0001

## Discussion

*BA* and *WS* are well-documented for their antihyperlipidemic properties [22]. The current research investigated the effects of *BA*, *WS*, and PHC on lipid profiles in T2DM Wistar rats. Our results reveal several key insights regarding how these intervention therapies affect the influence of lipid metabolism. A significant reduction in TC, LDL, and TG was observed in the BA, WS, and PHC-treated groups. The synergistic effect observed in the PHC group may be attributed to the combined actions of *BA* and *WS*, enhancing lipid metabolism through multiple pathways. This is consistent with the concept of PHC, which is designed to target multiple biochemical pathways simultaneously.

Moreover, the improvement in HDL levels in the treated groups suggests that these interventions not only reduce harmful lipid fractions but also enhance protective lipid components. This dual action is crucial in managing the dyslipidemia commonly associated with T2DM, which is a significant risk factor for cardiovascular diseases. The ability of these natural compounds to improve lipid profiles without adverse effects highlights their potential as complementary therapies in T2DM management.

Effect of *BA* on lipid profile

*BA* showed a significant impact on lipid profiles in diabetic rats, specifically a notable reduction in TC and LDL levels mentioned in Table 2. This reduction was observed across all tested doses (250, 500, and 1000 mg/kg body weight), with values comparable to those achieved with the standard antidiabetic drugs MET and GLI. Notably, the lipid-lowering effect of BA was significant, with LDL levels approaching those of the NC group, indicating its potential as a therapeutic agent for dyslipidemia associated with diabetes. Interestingly, *BA* did not significantly affect TG levels. This suggests that while *BA* has a strong influence on TC and LDL levels, its effect on TG may be limited or require higher doses to achieve a significant impact. These results are consistent with previous studies, which suggest that *Berberis* species have beneficial effects on lipid profiles. Berberine, a key component of *BA*, has been documented to lower TC and LDL while enhancing HDL levels in various animal models and clinical trials [23,24]. Modulation of lipid metabolism occurs by influencing key enzymes and pathways involved in cholesterol and TG regulation. Previous studies conducted by Caliceti et al. (2020) and Zhang et al. (2018) have demonstrated that berberine exerts lipid-lowering effects by inhibiting hepatic lipogenesis and promoting cholesterol excretion [25,26].

However, not all studies have reached the same conclusions. Some research suggests that the lipid-lowering effects of *Berberis* species, particularly berberine, might differ based on the specific conditions and models used in the experiments. For example, the study by Lee et al. in 2006 found that berberine did not significantly lower LDL levels in a different diabetic rat model, which might be due to variations in dosage, treatment duration, or the particular strain of rats involved. Similarly, Kong et al. in 2004 observed that while berberine was effective in reducing total cholesterol and LDL levels, it unexpectedly increased triglyceride levels under certain hyperlipidemic conditions. This highlights that berberine's impact on lipid metabolism may be more complex and dependent on the specific context [23,27]

Effect of *WS *on lipid profile

*WS* also demonstrated significant effects on lipid profiles similar to *BA*;* WS* administration led to a reduction in TC and LDL levels, as mentioned in Table 3. The reductions were evident across all dosages (250, 500, and 1000 mg/kg body weight), with the *WS* 1000 dose showing effects comparable to MET and GLI groups. However, *WS* treatment did not normalize TG levels to the same extent as TC and LDL, suggesting a selective impact on lipid metabolism. Moreover, *WS* effectively increased HDL levels, particularly at higher doses, which is consistent with its known benefits on cardiovascular health [28,29]. Previous research supports these findings, showing that *WS* can improve lipid profiles and reduce oxidative stress, which is beneficial for managing diabetes and associated dyslipidemia [30]. A report submitted by Mishra et al. in 2019 stated that *WS* is also well known for its role in reducing lipid peroxidation and improving antioxidant status, which collectively contributes to better lipid profiles in diabetic models [31].

While many studies support the benefits of *WS* on lipid profiles, it is important to acknowledge that not all research aligns with these findings. For example, the study conducted by Udaykumar et al. in 2009 observed that WS did not significantly influence lipid profiles in diabetic rats. They noted only slight reductions in TC and LDL levels at higher doses and found no significant effect on TG. This contrasts with our findings, where *WS* demonstrated selective effects on lipid metabolism. Similarly, a study by Bashir et al. in 2023 reported no impact of *WS* on HDL levels, raising questions about its cardiovascular benefits. These differences might be due to variations in experimental design, dosage, treatment duration, or the specific diabetic models used. Such inconsistencies highlight the need for further research to better understand the role of WS in lipid metabolism and its potential in diabetes management [32,33].

Effect of PHC on lipid profile

The PHC further enhanced the lipid profile modulation, as observed in Table 4. The PHC treatment led to significant reductions in TC and LDL levels and improvements in HDL levels across all the tested doses (250, 500, and 1000 mg/kg body weight). These effects were comparable to or superior to those observed with individual treatments and the standard antidiabetic drugs. The PHC treatment also had a notable impact on TG levels, reducing them significantly at all doses compared to the DC group. This suggests a synergistic effect of *BA* and *WS* in modulating lipid profiles more effectively than either herb alone. The enhanced lipid-lowering and HDL-boosting effects of the PHC may be attributed to the complementary mechanisms of action of the individual herbs. The results of our study are consistent with earlier research, which has shown that natural products, particularly those with antioxidant properties, can effectively modulate lipid profiles in diabetic models conducted by Sabu et al. in 2002 and Abhishek et al. in 2011 [34,35].

It is important to note that not all research agrees with our findings. For example, Reddy et al. found that a PHC containing *BA* and other herbal extracts did not significantly boost HDL levels in diabetic rats, contrasting with our observation of a notable increase in HDL. Similarly, Mikulska et al. discovered that while *WS* alone was effective at lowering TG, combining it with other herbs could not enhance this effect, which challenges our view of the PHC's ability to reduce TG levels. Additionally, Vinod et al. reported that a similar PHC had less impact on lowering LDL levels compared to standard T2DM medications [30,36,37].

These differences could stem from variations in the PHC, experimental methods, dosages, or the specific diabetic models used. Although our study highlights a significant benefit of *BA* and *WS*, it is important to recognize that the effectiveness of PHC can differ. More research is needed to resolve these inconsistencies and better understand the potential of these treatments.

Limitation

The study only used a single species of rats, i.e., albino Wistar rats. In our setup, we were unable to quantify the active principle present in the extract. The efficacy of the extract as a lipid-lowering agent can be further evaluated using other suitable animal models in different species and variants. The efficacy of the extract could be evaluated in human studies. The exact molecular mechanisms through which *BA*,* WS*, and PHC exert their lipid-lowering effects required further investigation.

## Conclusions

In summary, *BA* and *WS* both exhibit significant lipid profile modulation in T2DM Wistar rats, with *BA* mainly affecting TC and LDL levels and *WS* positively impacting HDL levels. The PHC of these two plants demonstrates superior efficacy in improving overall lipid profiles, including reductions in TC, LDL, and TG and increases in HDL. These findings suggest that *BA* and *WS*, particularly in PHC at a ratio of 1:1, hold potential as effective treatments for managing dyslipidemia in T2DM.

## References

[1] Tripathi KD (2019). Essentials of Medical Pharmacology. Jaypee Brothers Medical Publishers (P) Ltd.

[2] Goyal R, Singhal M, Jialal I (2023). Type 2 diabetes. StatPearls [Internet].

[3] Chakraborty S, Verma A, Garg R, Singh J, Verma H (2023). Cardiometabolic risk factors associated with type 2 diabetes mellitus: a mechanistic insight. Clin Med Insights Endocrinol Diabetes.

[4] Schofield JD, Liu Y, Rao-Balakrishna P, Malik RA, Soran H (2016). Diabetes dyslipidemia. Diabetes Ther.

[5] Feingold KR (2023). Dyslipidemia in patients with diabetes. Endotext [Internet].

[6] Daniel MJ (2011). Lipid management in patients with type 2 diabetes. Am Health Drug Benefits.

[7] (2024). The Calgary guide to understanding disease. https://calgaryguide.ucalgary.ca/content/.

[8] Belwal T, Bisht A, Devkota HP (2020). Phytopharmacology and clinical updates of Berberis species against diabetes and other metabolic diseases. Front Pharmacol.

[9] Sharma S, Chaitanya MVNL, Sharma S (2024). The medicinal plant berberis aristata and its endophytes for pharmacological applications: current research and future challenges. J Appl Biol Biotech.

[10] Rani R, Dahiya S, Dhingra D, Dilbaghi N, Kaushik A, Kim KH, Kumar S (2019). Antidiabetic activity enhancement in streptozotocin + nicotinamide-induced diabetic rats through combinational polymeric nanoformulation. Int J Nanomedicine.

[11] Modak M, Dixit P, Londhe J, Ghaskadbi S, Devasagayam TP (2007). Indian herbs and herbal drugs used for the treatment of diabetes. J Clin Biochem Nutr.

[12] Yedjou CG, Grigsby J, Mbemi A, Nelson D, Mildort B, Latinwo L, Tchounwou PB (2023). The management of diabetes mellitus using medicinal plants and vitamins. Int J Mol Sci.

[13] Salehi B, Ata A, V Anil Kumar N (2019). Antidiabetic potential of medicinal plants and their active components. Biomolecules.

[14] Bober Z, Stępień A, Aebisher D, Ożóg Ł, Bartusik-Aebisher D (2018). Fundamentals of the use of Berberis as a medicinal plant. Eur J Clin Exp Med.

[15] Rakha A, Ramzan J, Umar N (2023). The role of ashwagandha in metabolic syndrome: a review of traditional knowledge and recent research findings. J Biol Regul Homeost Agents.

[16] Zhou X, Seto SW, Chang D, Kiat H, Razmovski-Naumovski V, Chan K, Bensoussan A (2016). Synergistic effects of Chinese herbal medicine: a comprehensive review of methodology and current research. Front Pharmacol.

[17] Tiwari DD, Thorat VM, Pakale PV, Patil SJ (2024). Study of antidiabetic properties of Berberis asiatica and Withania somnifera in streptozotocin-nicotinamide-induced type ii diabetes mellitus in Wistar rats. Cureus.

[18] (2024). Test no. 240: Medaka extended one generation reproduction test (MEOGRT). https://doi.org/10.1787/9789264242258-en..

[19] Aamir K, Khan HU, Hossain CF (2022). Arjunolic acid downregulates elevated blood sugar and pro-inflammatory cytokines in streptozotocin (STZ)-nicotinamide induced type 2 diabetic rats. Life Sci.

[20] Chang W, Chen L, Hatch GM (2015). Berberine as a therapy for type 2 diabetes and its complications: from mechanism of action to clinical studies. Biochem Cell Biol.

[21] Sampson M, Ling C, Sun Q (2020). A new equation for calculation of low-density lipoprotein cholesterol in patients with normolipidemia and/or hypertriglyceridemia. JAMA Cardiol.

[22] Rana P, Kumar A, Choudhary A, Kaur H, Singh R (2021). The wisdom of prevention: holistic, preventive herb approach for healing of the globe. J Pharm Innov.

[23] Kong W, Wei J, Abidi P (2004). Berberine is a novel cholesterol-lowering drug working through a unique mechanism distinct from statins. Nat Med.

[24] (2024). The prevention of cruelty to animal act, 1960. https://ccsea.gov.in/Content/54_1_ACTS,RULESANDGUIDELINES.aspx.

[25] Caliceti C, Franco P, Spinozzi S, Roda A, Cicero FGA (2016). Berberine: new insights from pharmacological aspects to clinical evidences in the management of metabolic disorders. Curr Med Chem.

[26] Li L, Xiao Y, Zhou J (2024). Effects of Berberine on glucolipid metabolism among dehydroepiandrosterone-induced rats of polycystic ovary syndrome with insulin-resistance. Heliyon.

[27] Lee YS, Kim WS, Kim KH (2006). Berberine, a natural plant product, activates AMP-activated protein kinase with beneficial metabolic effects in diabetic and insulin-resistant states. Diabetes.

[28] Khandelwal KR (2008). Practical Pharmacognosy Techniques and Experiments. https://books.google.co.in/books?hl=en&lr=&id=SgYUFD_lkK4C&oi=fnd&pg=PA1&dq=Khandelwal,+K.+R.+(2004).+Practical+pharmacognosy+techniques+and+experiments.+Nirali+Prakashan.&ots=Sf9Ssoiz4N&sig=8leuEv4dOXkRdNyGOdNbTgbgwk0#v=onepage&q&f=false.

[29] Ibrahim M, Pawar G, Khan N, Gudalwar B, Khan S, Mazhar M (2024). Therapeutic effect of Withania somnifera (ashwagandha) on depression: a comprehensive review. NRFHH.

[30] Mikulska P, Malinowska M, Ignacyk M (2023). Ashwagandha (Withania somnifera)—current research on the health-promoting activities: a narrative review. Pharmaceutics.

[31] Mishra LC, Singh BB, Dagenais S (2019). Scientific basis for the therapeutic use of Withania somnifera (Ashwagandha): a review. Altern Med Rev.

[32] Udayakumar R, Kasthurirengan S, Mariashibu TS (2009). Hypoglycaemic and hypolipidaemic effects of Withania somnifera root and leaf extracts on alloxan-induced diabetic rats. Int J Mol Sci.

[33] Bashir A, Nabi M, Tabassum N, Afzal S, Ayoub M (2023). An updated review on phytochemistry and molecular targets of Withania somnifera (L.) Dunal (Ashwagandha). Front Pharmacol.

[34] Bhanot A, Sharma R, and Noolvi MN (2011). Natural sources as potential anti-cancer agents: a review. Phytomedicine.

[35] Sabu MC, Kuttan R (2002). Anti-diabetic activity of medicinal plants and its relationship with their antioxidant property. J Ethnopharmacol.

[36] Reddy KS, Sudheer A, Pradeepkumar B, Reddy CS (2019). Effect of a polyherbal formulation in streptozotocin-induced diabetic nephropathy in Wistar rats. Indian J Pharmacol.

[37] Gauttam VK, Kalia AN (2013). Development of polyherbal antidiabetic formulation encapsulated in the phospholipids vesicle system. J Adv Pharm Technol Res.

